# The information theory of individuality

**DOI:** 10.1007/s12064-020-00313-7

**Published:** 2020-03-24

**Authors:** David Krakauer, Nils Bertschinger, Eckehard Olbrich, Jessica C. Flack, Nihat Ay

**Affiliations:** 1 Santa Fe Institute, Santa Fe, USA; 2 Max Planck Institute for Mathematics in the Sciences, Leipzig, Germany; 3 Frankfurt Institute for Advanced Studies, Frankfurt am Main, Germany

**Keywords:** Shannon information, Mutual information, Information decomposition, Shared information, Synergy, Adaptation, Evolution, Control, Gestalt

## Abstract

Despite the near universal assumption of individuality in biology, there is little agreement about what individuals are and few rigorous quantitative methods for their identification. Here, we propose that individuals are aggregates that preserve a measure of temporal integrity, i.e., “propagate” information from their past into their futures. We formalize this idea using information theory and graphical models. This mathematical formulation yields three principled and distinct forms of individuality—an organismal, a colonial, and a driven form—each of which varies in the degree of environmental dependence and inherited information. This approach can be thought of as a Gestalt approach to evolution where selection makes figure-ground (agent–environment) distinctions using suitable information-theoretic lenses. A benefit of the approach is that it expands the scope of allowable individuals to include adaptive aggregations in systems that are multi-scale, highly distributed, and do not necessarily have physical boundaries such as cell walls or clonal somatic tissue. Such individuals might be visible to selection but hard to detect by observers without suitable measurement principles. The information theory of individuality allows for the identification of individuals at all levels of organization from molecular to cultural and provides a basis for testing assumptions about the natural scales of a system and argues for the importance of uncertainty reduction through coarse-graining in adaptive systems.

## The architecture of individuality

From the perspective of physics and chemistry, biological life is surprising. There is no physical or chemical theory from which we can predict biology, and yet if we break down any biological system into its elementary constituents, there is no chemistry or physics remaining unaccounted for (Gell-Mann 1995). The fact that physics and chemistry are universal—ongoing in stars, solar systems, and galaxies—whereas to the best of our knowledge biology is exclusively a property of earth, supports the view that life is emergent. This stands in contrast to the universality of chemical phenomena which can be predicted from quantum mechanical considerations in fundamental physics even when this proves to be computationally cumbersome or intractable (Defranceschi and Le Bris 2000). The asymmetry in what can be gleaned from working down toward ever more elementary constituents versus working up through levels of aggregation is captured by the terms reductionism and emergence (Anderson 1972; Laughlin and Pines 2000). It is often difficult to predict physical properties of aggregates from knowledge of constituents, and this extends to questions of behavior where it is rarely clear how far “down” to go (Anderson 1972; Krakauer and Flack 2010a; Flack 2017b). There are assumed to be dominant microscopic scales for a given set of aggregate properties yet our understanding of what constitutes a fundamental unit (Gilbert et al. 2012; Daniels et al. 2016) and whether these units count as individuals, have implications for many areas of science, from taxonomy and cladistics through to physiology, behavior, and ecology (Clarke 2011; Wilson and Barker 2013).

It is almost inconceivable for us to imagine a biological science without a concept of units or individuality. After all, how could we speak about metabolism, behavior or the genome without first establishing a unit or container of observation and measurement? Even Schrödinger in his prescient book, *What is Life?* (Schrodinger 2012), sought to explore the persistence of biological phenotypes of organisms—or even features of ecosystems—through the lens of elementary and universal physical underpinnings, made strong prior assumptions about the reality of individual organisms:

“What degree of permanence do we encounter in hereditary properties and what must we therefore attribute to the material structures which carry them? The answer to this can really be given without any special investigation. The mere fact that we speak of hereditary properties indicates that we recognize the permanence to be of the almost absolute. For we must not forget that what is passed on by the parent to the child is not just this or that peculiarity...Such features we may conveniently select for studying the laws of heredity. But actually it is the whole (four- dimensional) pattern of the phenotype, all the visible and manifest nature of the individual, which is reproduced without appreciable change for generations, permanent within centuries—though not within tens of thousands of years—and borne at each transmission by the material in a structure of the nuclei of the two cells which unite to form the fertilized egg cell. That is a marvel.”

Schrödinger did not set out to derive the individual from fundamental physics but to reconcile existing and rather traditional conceptions of individuality (essentially the individual as synonymous with the observable organism) with the new physics of quantum mechanics.

In this respect, Schrödinger was adopting a typically reductionist perspective, explaining features of biological science through first principles of physics (Weinberg 1995). In Schrödinger’s case, the physical feature of greatest importance to biology was the long-lived covalent bond. But for many reasons this line of approach has failed to deliver the deep and unifying insights based on physics (Anderson 1972), from which powerful biological ideas—such as adaptation or individuality—might be derived (Dupré 2009; Keller 2009).

The question we seek to address is more limited. How do we identify individuals without relying on features like cell membranes that may be solutions to challenges faced by particular systems for maintaining integrity rather than foundational properties? We want to allow for the possibility that microbes and loosely bound ecological assemblages such as microbial mats and cultural and technological systems, when viewed with a mathematical lens, qualify as individuals even though their boundaries are more fluid than the organisms we typically allow. It may also be the case that entities currently considered individuals are indeed individuals but not in the way we think—organisms are more complicated than typical individuality definitions acknowledge. Humans for example contain approximately as many self-cells as symbiotic microbes (Andreu-Moreno and Sanjuán 2018), yet until recently with the advent of the concept of “holobiont” (Gilbert et al. 2012), the microbe portion of the human cellular ecosystem was not typically considered part of the human individual.

In an ideal case, visitors to an exoplanet would have a procedure for identifying or “perceiving” individuals based on a quantitative survey with minimal prior knowledge of the type of life form that they expect to encounter. In the next sections of the paper, we briefly review a few key standard assumptions about individuality in biology and challenges to formalizing the concept. We then discuss a way forward and develop an information-theoretic formalism.

### Standard assumptions and challenges

Here, we briefly review some of the criteria currently used to identify individuals. For a synoptic treatment of individuality definitions see (Clarke 2011; Gilbert et al. 2012). A standard assumption is that replication presupposes individuality (Wilson and Barker 2013). Under this assumption, replicators typically include organisms that have developed from a fertilized egg, with individuality residing at the phenotypic level (see Dawkins 1983), and asexual microbes or clonal organisms for which individuality is defined based on shared genetic ancestry (Hughes 1990). The replicator assumption has served as the starting point for theorizing about what an individual is in a broad class of studies and out of this work has come three additional widely accepted properties of biological individuals: (1) they can increase in relative frequency by exploiting a source of metabolic free energy, (2) they respond adaptively to their environments, and (3) they are characterized by tightly coordinated relationships (chemical, physiological, computational) among their parts. The association of these properties with individuality has raised debate about whether individuality applies only to “single” organisms, as the replicator assumption suggestions, or also to cells and aggregates like societies (Gilbert et al. 2012).

Beyond replicators as proxies for an individuals, almost all definitions of individuality assume a set members (individual) and a set complement (environment). These are articulated in different ways including: (1) as an immunological concept pertaining to the idea of self and non-self (Pradeu 2012), (2) as a temporal aggregate encoding a common past separable or independent from the past of other aggregates (ontogenetic or phylogenetic) (Rieppel 2013) (3) as a spatially bounded collection of metabolic reactions insulated by a membrane from reactions in the environment (Rasmussen et al. 2004), and more abstractly, (4) as a unit of selection and evolutionary change (Buss 1987; Hughes 1990; Szathmáry and Smith 1997; Callcott and Sterelny 2011).

To reveal limitations of the above definitions and other hidden assumptions, a useful exercise is to consider aggregates and processes that do not typically get classified as individuals (Santelices 1999).

Work on social insects and on a number of plant, fungal and prokaryotic species demonstrates the possibility of *individuality simultaneously at multiple organizational levels*—physically distinct ants form aggregations called colonies and these colonies may be divided into spatially noncontiguous subsets (Gow et al. 2008; Esser et al. 2001). Furthermore, in many ant species the majority of worker ants do not replicate and the colony as whole does not replicate, but contiguity between past and future is nonetheless a feature of the system. And, importantly, it is the combination of reproduction by a minority of colony members coupled to the industry of the majority that allows the colony as a whole to adapt in response to changes in the environment. Taken together, these two observations suggest *it is possible to have individuality without replication and some forms of individuality benefit when replication is partial*.

Viruses occupy a figurative twilight zone in biology. Declared by some non-living, and treated by most as a rather pathetic minimal limit of life, viruses constitute obligate translational parasites, incapable of completing their life cycles without first appropriating the protein synthesis machinery of a host cell. The viral capsid contains a largely inert genome responsible for encoding only a small fraction of the proteins required for synthesizing a new virus genome and the capsid required for egressing from the infected cell. The virus exists only within the larger dynamical, regulatory network of the cell. Hence, the virus—understood as the active parasitic agent—is comprised largely of host encoded factors. And yet it can replicate, adapt, and has a persistent identity that distinguishes it from its “host” environment—despite the fact it relies on its “host” environment for replicating. And, recent work suggests that viruses like microbes form collective units that facilitate infection (Andreu-Moreno and Sanjuán 2018). These observations suggest that viruses in aggregate are individuals but not in the conventional sense. Rather they are what Krakauer (Krakauer and Zanotto 2006) has called “chimerical individuals.”

### A way forward

The above examples are fascinating but without a rigorous definition of both the environment and the agent it is difficult to speak consistently of individuals. This is analogous to figure-ground separation in gestalt psychology or computer vision. The background of an image carries as much if not more information than the object, and the challenge is to separate the two rather than assume that they are already distinct and independent.

One possibility is that ant colonies and viruses [or humans, for that matter, composed of 37 trillion microbes and 30 trillion “human” cells (Andreu-Moreno and Sanjuán 2018; Gilbert et al. 2012)] are only nominally individual—a categorization resulting from human perceptual bias for certain kinds of aggregation. But if they are real in a deeper physical sense then how might we determine this? We propose:

- Individuality can be *continuous*, with the possible surprising result that some processes possess greater individuality than others.
- Individuality can emerge at any level of organization. This requires we dispense with privileging a single level or object—for example, replicating cells or organisms—and then defining individuality based on features of these objects, such as sequestered germ cells, vertical transmission of genetic material, a common pool of metabolic free energy, or coordinated immune responses. Although these features may indeed be effective proxies when we have significant prior knowledge of a system, *our goal should be to find fundamental, rather that derivative, properties of individuality. Defining individuality around derived properties risks precluding the possibility of individuality in at super-organismal levels and in distributed systems*.
- Individuality can be nested. Given that life is hierarchically organized into trophic and functional levels, we allow the possibility of multiple, parallel levels of individuality. We take this position to be related to the recent suggestion of (Rieppel 2013) where he argues for individuals based on hierarchical complexes of homeostatic properties and (Flack 2017a) who has proposed biological systems are information hierarchies resulting from the collective effects of components estimating, in evolutionary or ecological time, regularities in their environments by coarse-graining or compressing time series data and using these perceived regularities to tune strategies. As coarse-grained (slow) variables become for components better predictors than microscopic behavior (which fluctuates), and component estimates of these variables converge, new levels of organization consolidate.

Allowing individuality to be continuous rather than binary, nested, and possible at any level, opens the door for more quantitative takes on familiar open questions in evolutionary theory including the relation between the units of selection and temporal and spatial correlation and whether individuality at one scale impacts coherence and autonomy and “lower” and “higher” scales. These revealed time and space scales and their interdependencies should provide clues about the mechanisms driving their consolidation and through this consolidation the emergence of individuals (Flack 2017a, b).

Given our proposition that individuals are aggregates that “propagate” information from the past to the future and have temporal integrity, and that individuality is a matter of degree, can be nested, distributed and possible at any level, how can we formalize individuality?

## Formalizing individuality

We will take as our starting point measurements from a stochastic process. This could be a vector of chemical concentrations over time, the abundance of various cell types, or probabilities of observing coherent behaviors. We use coarse-grained or quantized information-theoretic filters the quantize the measurements. Some of these filters will reveal a coordinated pattern of behavior, whereas others will filter out all signal and detect nothing. Thus, signal amplitude given an appropriate filter becomes a means of discovering different forms of individuality. This is somewhat analogous to observing patterns in infrared that would be invisible using the wavelengths of visible light—individuality is revealed through characteristic patterns of information flow.

The basis for this approach to aggregation comes from information theory, and throughout this paper we assume that individuals are best thought of in terms of dynamical processes and not as stationary objects that leave information-theoretic traces. In this respect, our approach might reasonably be framed through the lens of “process philosophy” (Rescher 2007) which makes the elucidation of the dynamical and coupled properties of natural phenomena the primary explanatory challenge. From the perspective of “process philosophy,” the tendency of starting with objects and then listing their properties—“substance metaphysics”—places the cart before the horse.

### The origin of information

Our proposal that individuals are aggregates that propagate information from the past to the future and have temporal integrity can be viewed as a pragmatic operational definition that captures the idea there is something persistent about individuals. However, our motivation for defining individuality this way is actually much deeper. It lies in the information-theoretic interpretation of entropy, its connection to the physical theory of thermodynamics, and formal definition of work introduced by Clausius in the 1860s [see (Müller 2007) for an introduction to this history].

Briefly, work (displacement of a physical system) is produced by transferring thermal energy from one body to another (heat). Entropy captures, or measures, the loss in temperature over the range of motion of the working body. In other words, entropy measures the energy lost from the total available energy available for performing work. The insights of Clausius were formalized and placed in a mathematical framework by Gibbs in 1876.

In 1877, Boltzmann provided in his kinetic theory of gasses an alternative interpretation of entropy. For Boltzmann entropy is a measure of the potential disorder in a system. This definition shifts the emphasis from energy dissipated through work to the number of unobservable configurations (microstates) of a system, e.g., particle velocities consistent with an observable measurement (macrostate), e.g., temperature. The thermodynamic and Boltzmann definitions are closely related as Boltzmann entropy increases following the loss of energy available for work attendant upon the collision of particles in motion during heat flow. There are many different microscopic configurations of individual particles compatible with the same macroscopic measurement, and only a few of which are useful.

In 1948, encouraged by John von Neumann, Claude Shannon used the thermodynamical term entropy to capture the information capacity of a communication channel. A string of a given length (macrostate) is compatible with a large number of different sequences of symbols (microstates). A target word will be disordered during transmission in proportion to the noise in a channel. If there were no noise, each and every microstate could be resolved and the entropy would define an upper limit on the number of signals that could be transmitted. The study of the maximum number of states that can be transmitted from one point to another across a channel, in the face of noise and when efficiently encoded, is called information theory.

Shannon did not describe entropy in terms of heat flow and work but in terms of information shared through a channel transmitted from a signaler to a receiver. The power of information theory derives in part from the incredible generality of Shannon’s scheme. The signaler can be a phone in Madison and the receiver a phone in Madrid, or the signaler can be a parent and the receiver its offspring. For phones, the channel is a fiber-optic cable and the signal pulses of light. For organisms the channel is the germ line and the signal the sequence of DNA or RNA polynucleotides in the genome. Increasing entropy for a phone-call corresponds to the loss or disruption of light-pulses, whereas increasing entropy during inheritance corresponds to mutation or developmental noise. The same scheme can be applied to development, in which case the signaler is an organism in the past and the receiver the same organism in the future. One way in which we might identify individuals is to check to see whether we are dealing with the same aggregation at time *t* and $t+1$. *If the information transmitted forward in time is close to maximal, we take that as evidence for individuality*.

In its simplest form, Shannon made use of the following formal measures when defining information. The entropy *H* of a random variable *S* measures the uncertainty or information of the states that it can adopt:

$$\begin{aligned} H(S) = -\sum _i P(s_i)\log _2 P(s_i) \end{aligned}$$

where $s_i$ are the possible values of the state and $P(s_i)$ the probabilities of these states. For a coin there would be two possible values for *S*, heads and tails, and the values of these states for a fair coin would be the probability 0.5, yielding a metric entropy value of 1. Deviation from a fair coin corresponds to a reduction in information, as in the limit of bias where only one side of the coin is favored, the outcome is known in advance and any toss of the coin is perfectly predictable. This produces an entropy value of 0. Hence, information is minimized when predictability is maximized.

To capture the communication value of information Shannon introduced a signaler–receiver structure, which is now typically described using two random variables *S* and *R*. The maximum information transmitted between signaler and receiver is given by the Mutual Information (*I*). The *I* can be written in several different forms. One intuitive expression is:

$$\begin{aligned} I(S;R) = H(S) + H(R) - H(S,R) \end{aligned}$$

where *H*(*S*) and *H*(*R*) are the entropies of the signals, and *H*(*S*; *R*) the joint entropy of the two variables,

$$\begin{aligned} H(S;R) = -\sum _i \sum _j P(s_i, r_j)\log _2 P(s_i, r_j) \end{aligned}$$

The joint entropy is at a maximum when there is no relationship between the *S* and *R* variables. The *I* is therefore high when the information in *S* and *R* are high and they are strongly coupled in their values (*H*(*S*; *R*) is low). The *I* measures the information shared between *S* and *R* over a communication channel, because the only source of structure in *R* is assumed to come from *S*.

Another conventional way of writing *I* is,

$$\begin{aligned} I(S;R) = H(R) - H(R|S) \end{aligned}$$

where *H*(*S*|*R*) is the conditional entropy of *R* or the amount of information in *R* that is not in *S*. Hence, if all the information in *R* comes from *S* then *H*(*R*|*S*) will be zero, and $I(S;R) = H(R)$. If one of the random variables, for example the sender *S* consists of two parts $S=\{S_1,S_2\}$, we can decompose the mutual information using the chain rule (Cover and Thomas 1991)

$$\begin{aligned} I(S_1,S_2;\,R)=I(S_1;\,R)+I(S_2;\,R|S_1) \end{aligned}$$

with the second term being the conditional mutual information

$$\begin{aligned} I(S_2;\,R|S_1):=H(R|S_1)-H(R|S_1,S_2). \end{aligned}$$

These measures provide the necessary statistics for an informational theory of the individual.

**Fig. 1:**
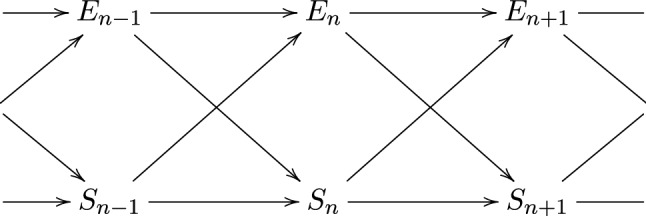
The causal diagram of the system–environment interaction

When we model the interaction between a system and its environment we have to consider a more complicated situation which involves two channels. To be more precise, let ${{\mathcal {S}}}$ and ${{\mathcal {E}}}$ be the state set of the system and the environment. For simplicity, we assume that ${{\mathcal {S}}}$ and ${{\mathcal {E}}}$ are finite. The dynamics of the system is influenced by its own state, but it can also be influenced by the state of the environment. This can be modeled in terms of a channel $\varphi : {{\mathcal {E}}} \times {{\mathcal {S}}} \rightarrow {{\mathcal {S}}}$, where $\varphi (e,s;s')$ denotes the probability of the next system state $s'$ given that the current system state is *s* and the environment is in state *e*. In particular, we assume that $\varphi (e,s;s') \ge 0$ for all $e,s,s'$, and $\sum _{s'} \varphi (e,s;s') = 1$ for all *e*, *s*. We can model the dynamics of the environment in the same way, using a Markov kernel $\psi : {{\mathcal {S}}} \times {{\mathcal {E}}} \rightarrow {{\mathcal {E}}}$, where $\psi (s,e;e')$ denotes the probability for the next state $e'$ of the environment given the current states *e* and *s* of the environment and the system, respectively. The kernels $\varphi$ and $\psi$ model the mechanisms that constitute the system–environment interaction. If we start this interaction process by selecting a states *s* and *e* according to some probability distribution $\mu$, we obtain a process $(S_k,E_k)$, $k = 1,2,\ldots$, in ${{\mathcal {S}}} \times {{\mathcal {E}}}$ that satisfies

$$\begin{aligned}&{\mathbb P}(S_1 = s_1, E_1 = e_1, S_2 = s_2, E_2 = e_2, \ldots , S_n \\&\quad = s_n, E_n = e_n) \\&\quad = \mu (s_1,e_1) \, \varphi (e_1,s_1;s_2) \psi (s_1,e_1;e_2) \, \ldots \, \\&\qquad \varphi (e_{n - 1} , s_{n - 1} ; s_n) \psi (s_{n - 1} , e_{n - 1} ; e_n), \qquad n = 1, 2, \ldots . \end{aligned}$$

Clearly, we can recover the mechanisms from the distribution of the process $(S_k, E_k)$, $k = 1,\ldots ,$

$$\begin{aligned} {\mathbb P}(S_1&= {} s, E_1 = e) = \mu (s,e), \\ {\mathbb P}(S_k&= {} s' \, | \, E_{k - 1} = e, S_{k - 1} = s) = \varphi (e , s ; s' ), \\ {\mathbb P}(E_k &= {} e' \, | \, S_{k - 1} = s, E_{k - 1} = e) = \psi (s , e ; e' ). \end{aligned}$$

We apply information-theoretic quantities, such as the mutual information, to variables of the process $(S_k, E_k)$, thereby quantifying information flows between the system and the environment. The causal structure of the process, as shown in Fig. 1, implies a number of conditional independence statements. For instance $E_{n + 1}$ is conditionally independent of $S_n, E_n$ given $S_{n-1}, E_{n-1}$.

### The informational individual

In the previous section, we set down the information-theoretic foundations for our formalism. Here, we discuss the additional mathematical properties required of the formalism if it is to capture the concept of individuality we developed in “A way forward” section.

We remind the reader our starting point is the assumption that biological individuality can usefully be understood as an “informational individual.” We further remind the reader this is not to be confused with Dawkin’s replicator, as we want to allow the possibility that replication is not a fundamental feature of individuality and be able to ask what role individuality plays in facilitating replication. What *is* fundamental in our view is the idea that information can be propagated forward through time, meaning that uncertainty is reduced over time. In this way, and returning to our opening remarks in “A way forward” section, we suggest individuality is a natural extension of the ideas of Boltzmann and Von Neumann, and as such has foundations in statistical mechanics and thermodynamics, which consider the conditions required for a persistently ordered states.

Defining properties and implications of the formalism

1. *The system environment decomposition* Consider a dynamical set of quantifiable measurements that we coarse grain into components of a system and components of an environment. We seek a way of establishing whether this partition is justifiable, and whether the individuality concept is relevant. We wish to allow for a hierarchy of such partitions in order to capture biological examples such as organelles within cells, and cells within bodies within populations, where in each case the target entity and the environment assume a different identity. We retain those partitions that meet our information-theoretic inclusion criteria and then can ask which among the natural, intuitive categories of biology—e.g., cells, organelles, organisms, populations, etc., are recovered.
2. *Informational individuals* In the pursuit of generality, we consider a discrete, stochastic process where the state of the system in the future is determined by some subset of states in the present. If we arbitrarily divide these states into system and environment, we should like to be able to determine how the current system state $S_n$ and the current state of the environment $E_n$ together are sufficient to determine the next system state $S_{n+1}$. Formally, the predictability of the next state of the system is quantified via the mutual information:
  $$\begin{aligned} I(S_n, E_n; \,S_{n+1}) = H(S_{n+1}) - H(S_{n+1} | S_n, E_n) . \end{aligned}$$
  This expression seeks to capture how much information at time $$n+1$$$$S_{n+1}$$ comes from the system itself at a previous time step (or generation) $$S_n$$—the individual—versus from the environment at a previous time $$E_n$$. This mutual information can now be decomposed in two ways
  $$\begin{aligned} I(S_n, E_n; S_{n+1})&= {} I(S_{n+1}; S_n) + I(S_{n+1}; E_n | S_n) \\&= {} I(S_{n+1}; E_n) + I(S_{n+1}; S_n | E_n) \end{aligned}$$
  Each decomposition can be interpreted as different allocation for distributing the observed past regularities between the system and environment. Each of these will allow us to define different forms of individuality.
  a *Endogenous determination* Consider $I(S_{n+1}; S_n) + I(S_{n+1}; E_n | S_n)$:
    Here, we measure the influence of the system state onto itself (at the next generation or time step). For a preferred interval of time, all observed dependencies between successive system states are attributed to the system.
    The quantity $I(S_{n+1}; S_n)$ has been called autonomy in Krakauer and Zanotto (2006) and will be denoted as $A^*$ in the following. It should be high when the system is largely control of its environment.
    The influence of the environment, as measured by $I(S_{n+1}; E_n | S_n)$, can be interpreted as new information for the system flowing from the environment into the system. When this information flow vanishes completely, a system can be said to be *informationally closed*. So this quantity measures the degree to which the system is controlled by the environment *nC*. Note that closure does not require causal independence, it only states that all influences from the environment are predictable by the system.
  b *Environmentally driven* An alternative to endogenous determination is structure imposed largely through environmental gradients driving the system. In other words, the history of the system is not as consequential as the history of the environment that impose strong boundary conditions on the system. Consider $I(S_{n+1}; E_n) + I(S_{n+1}; S_n | E_n)$:
    Here, the observed influences are attributed to the environment (as far as possible according to $I(S_{n+1}; E_n)$). Only the remaining influence $I(S_{n+1}; S_n |E_n)$ is due to the system. This can be interpreted as an alternative concept of system *autonomy* (Bertschinger et al. 2008) and will be denoted as *A* in the following. It is valid under the assumption that all dependencies between the states of the system and the environment are attributed to the environment.
  These properties allow us to identify three quantities, each corresponding to a type of individuality:
  $$\begin{aligned} \text{ Colonial } \text{ Individuality } \quad A&:=I(S_{n+1};S_n|E_n) \\ \text{ Organismal } \text{ Individuality } \quad A^{*}&:=I(S_{n+1};S_n) \\ \text{ Environmental } \text{ Determined } \text{ Individuality } \quad nC&:=I(S_{n+1};E_n|S_n) \end{aligned}$$
  To rigorously formalize these different types of individuality, however, we need to consider them on a more fine-grained scale.

### Fine-grained decomposition

Using the chain rule for mutual information, we encounter an ambiguity in attributing influence to the environment or to the system. The partial information decomposition (Williams and Beer 2010; Bertschinger et al. 2013). allows us to resolve this ambiguity by introducing notions of unique, shared and complementary information.^1^

The mutual information between the future state of the system at time $n+1$ and the joint state of system and environment at time *n* is decomposed into four terms:

1 $$\begin{aligned} I(S_{n+1};S_n,E_n)&= \underbrace{SI(S_{n+1};S_n,E_n)}_{\text {shared}} + \underbrace{CI(S_{n+1}; S_n,E_n)}_{\text {complementary}} \\&\quad+ \underbrace{UI(S_{n+1};S_n\backslash E_n)}_{\text {unique }(S_n\text { wrt }E_n)} + \underbrace{UI(S_{n+1};E_n\backslash S_n)}_{\text {unique }(E_n\text { wrt } S_n)}. \end{aligned}$$

Those four terms appear in the pairwise mutual information and conditional mutual information that we obtained from the chain rule:

2 $$\begin{aligned} I(S_{n+1};S_n)&= SI(S_{n+1};S_n,E_n) + UI(S_{n+1};S_n\backslash E_n) , \end{aligned}$$

3 $$\begin{aligned} I(S_{n+1};E_n|S_n)&= CI(S_{n+1};S_n,E_n) + UI(S_{n+1};E_n\backslash S_n) , \end{aligned}$$

4 $$\begin{aligned} I(S_{n+1};E_n)&= SI(S_{n+1};S_n,E_n) + UI(S_{n+1};E_n\backslash S_n) ,\end{aligned}$$

5 $$\begin{aligned} I(S_{n+1};S_n|E_n)&= CI(S_{n+1};S_n,E_n) + UI(S_{n+1};S_n\backslash E_n) , \end{aligned}$$

In our context the four terms have the following meaning:

a The unique information from the system $UI(S_{n+1};S_n \setminus E_n)$. This is information maintained by the system.
b The shared information between the system and environment $SI(S_{n+1},S_n,E_n)$.
c The unique information from the environment $UI(S_{n+1};E_n \setminus S_n)$. This quantifies the influence of the environment on the system. (Information flow in the narrow sense).
d The complementary or synergistic information. Information that is only present in the interaction of systems and environment.

It is important to emphasize that these decompositions are a means of supporting our formal intuition and do not correspond to a specification of the information-theoretic quantities. This choice remains disputed and several alternative proposals have been published. These are reviewed in a special issue of the journal Entropy (Lizier et al. 2018). Nevertheless, the measures that we derive fully accord with the conceptual decomposition.

### Forms of individuality

With a good understanding of the implications of partial information decomposition in hand in hand, we can now rigorously define three forms of individuality and an additional measure quantifying contribution of each in the case of hybrid types. These measures are defined in terms of the information that is shared by system and environment (e.g., adaptive information), information that is unique to either the system or the environment (e.g., memory in each), and information that depends in some complicated way on both the system and the environment (e.g., regulatory information).

- **Organismal Individuality**$$A^*$$
  $$\begin{aligned} A^{*}=SI(S_{n+1};S_n,E_n) + UI(S_{n+1};S_n\backslash E_n) \end{aligned}$$
  Organisms are well adapted when they share through adaptation or learning significant information with the environment in which they live. In addition, they contain a large amount of private information required for effective function. By maximizing this measure, we are able to identify complex organisms in their environments.
- **Colonial Individuality***A*
  $$\begin{aligned} A=CI(S_{n+1};S_n,E_n) + UI(S_{n+1};S_n\backslash E_n) \end{aligned}$$
  Many organisms such as microbes share only a small amount of information with the environment in which they live. They contain regulatory mechanisms that allow for adaptation through ongoing interaction between their biotic and abiotic environment. By maximizing this measure, we are able to identify “environmentally regulated aggregations,” which we call “colonial individuals.”
- **Environmental determination***nC*
  $$\begin{aligned} nC&=  {} I(S_{n+1};E_n|S_n) = CI(S_{n+1};S_n,E_n) \\&\quad + UI(S_{n+1};E_n\backslash S_n) \end{aligned}$$
  This measure quantifies the degree of environmental determinism on the temporal evolution of an individual. When this measure is minimized an individual becomes completely insensitive to the environment—and hence is neither in the organismal or colonial form—and not in any real sense adaptive. It represents the persistence of an environmental memory capable through interaction with the system of generating structure, such as temperature gradients in a fluid that produce vortices.
- **Environmental Coding**
  $$\begin{aligned} NTIC=SI(S_{n+1};S_n,E_n) - CI(S_{n+1};S_n,E_n) \end{aligned}$$
  The intuition behind this measure is to quantify the difference between a colonial and organismal measure of individuality. The difference is captured by the difference between shared information (e.g., adaptive information) and the interaction of individual and environment (e.g., regulatory information). One way to think about this is how much information can be encoded about the environment in the system innately (e.g., inherited information) versus how much information needs to be encoded through ongoing interaction. When the measure is large nature dominates nurture. As the measure declines, nurture begins to dominate nature.

### Individuality measures in an illustrative example

To gain a better understanding of each of these measures, we work through a quantitative example.

We consider two binary units $E_n$ and $S_n$, with state sets $\{-1, + 1\}$. Following the general structure introduced in sect. 2.1 and Fig. 1, these states are synchronously updated according to the following conditional distribution:

$$\begin{aligned} p(s_{n+1},e_{n+1} | s_n,e_n) \, = \, p_S(s_{n+1} | s_n, e_n) \cdot p_E(e_{n+1} | s_n,e_n), \end{aligned}$$

where

6 $$\begin{aligned} p_S(s_{n+1} | s_n, e_n)= {} \frac{1}{1 + \mathrm{e}^{- 2 s_{n+1} \left( \delta _S + \alpha _S s_n + \beta _S e_n + \gamma _S s_n e_n\right) }} \end{aligned}$$

7 $$\begin{aligned} p_E(e_{n+1} | s_n, e_n)= \frac{1}{1 + \mathrm{e}^{- 2 e_{n+1} \left( \delta _E + \alpha _E e_n + \beta _E s_n + \gamma _E s_n e_n\right) }}. \end{aligned}$$

Evaluating the individual conditional distributions, we obtain

$$\begin{aligned} p_S(+1 | +1, +1)&= \frac{1}{1 + \mathrm{e}^{- 2 \left( \delta _S + \alpha _S + \beta _S + \gamma _S \right) }} =: a_S \\ p_S(+ 1 | -1, +1)&= \frac{1}{1 + \mathrm{e}^{- 2 \left( \delta _S - \alpha _S + \beta _S - \gamma _S \right) }} =: b_S \\ p_S(+ 1 | +1,-1)&= \frac{1}{1 + \mathrm{e}^{- 2 \left( \delta _S + \alpha _S - \beta _S - \gamma _S \right) }} =: c_S \\ p_S(+ 1 | -1,-1)&= \frac{1}{1 + \mathrm{e}^{- 2 \left( \delta _S - \alpha _S - \beta _S + \gamma _S \right) }} =: d_S \end{aligned}$$

and correspondingly

$$\begin{aligned} p_E(+1 | +1, +1)&= {} \frac{1}{1 + \mathrm{e}^{- 2 \left( \delta _E + \alpha _E + \beta _E + \gamma _E \right) }} =: a_E \\ p_E(+ 1 | -1, +1)&= {} \frac{1}{1 + \mathrm{e}^{- 2 \left( \delta _E + \alpha _E - \beta _E - \gamma _E \right) }} =: b_E \\ p_E(+ 1 | +1,-1)&= {} \frac{1}{1 + \mathrm{e}^{- 2 \left( \delta _E - \alpha _E + \beta _E - \gamma _E \right) }} =: c_E \\ p_E(+ 1 | -1,-1)&= {} \frac{1}{1 + \mathrm{e}^{- 2 \left( \delta _E - \alpha _E - \beta _E + \gamma _E \right) }} =: d_E \end{aligned}$$

Finally, this yields the following stochastic matrix with entries $p(s_{n+1},e_{n+1} | s_n,e_n)$:

$$\begin{aligned} \begin{array}{ c || c | c | c | c |} &{} (+ 1, + 1) &{} (- 1, + 1) &{} (+ 1, -1) &{} (-1 , -1) \\ \hline \hline (+ 1, + 1) &{} a_S a_E &{} (1 - a_S) a_E &{} a_S (1 - a_E) &{} (1 - a_S) (1 - a_E) \\ (- 1, + 1) &{} b_S b_E &{} (1 - b_S) b_E &{} b_S (1 - b_E) &{} (1 - b_S) (1 - b_E) \\ (+ 1, - 1) &{} c_S c_E &{} (1 - c_S) c_E &{} c_S (1 - c_E) &{} (1 - c_S) (1 - c_E) \\ (- 1, - 1) &{} d_S d_E &{} (1 - d_S) d_E &{} d_S (1 - d_E) &{} (1 - d_S) (1 - d_E) \\ \hline \end{array} \end{aligned}$$

**Fig. 2:**
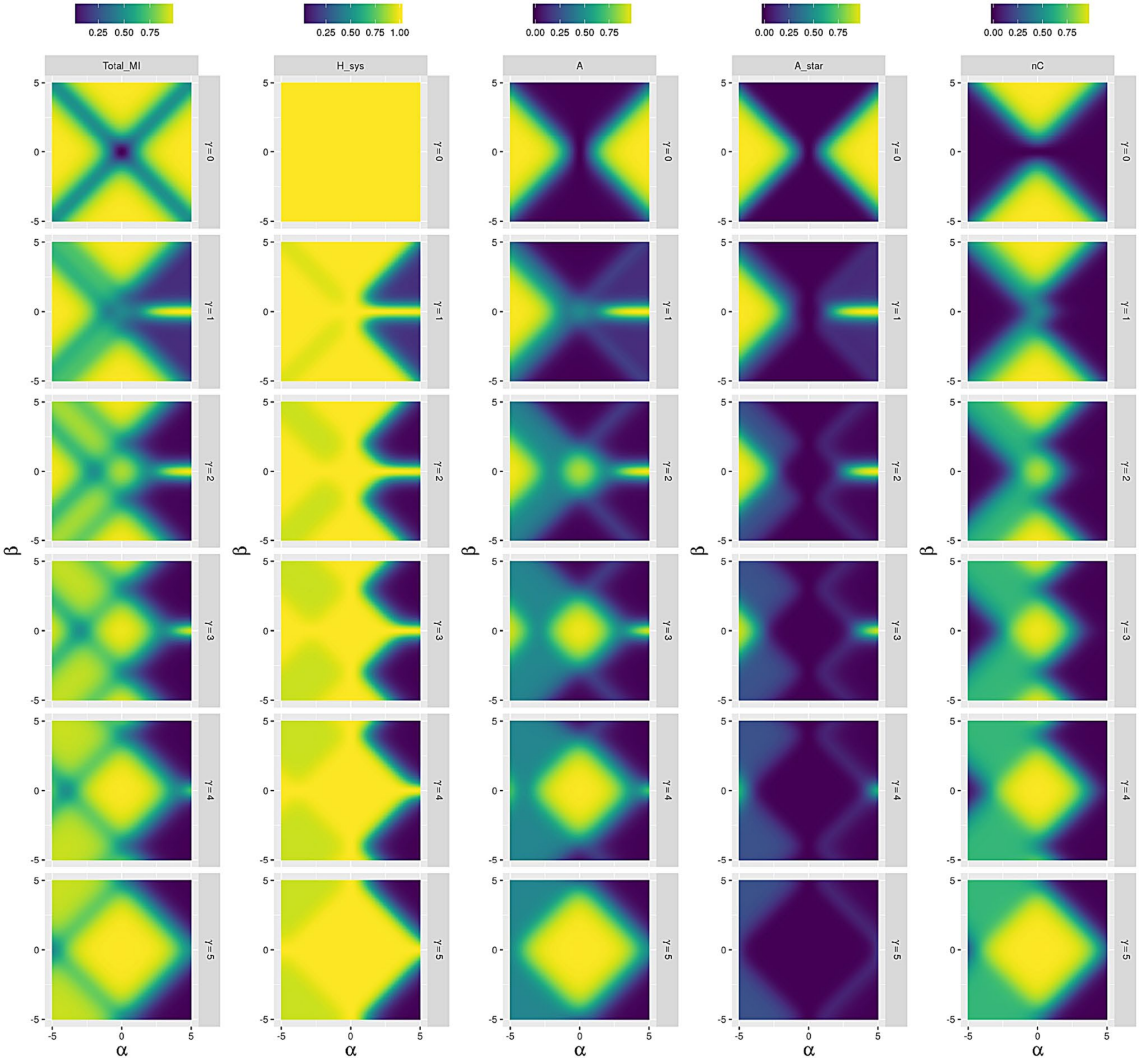
Mutual information between two time steps (Total_MI), Entropy of the system (H_sys), colonial (A) and organismal (A_star) individuality, and environmental determination (nC) for different values of $\alpha _S$,$\beta _S$, and for $\gamma _S$ (subscript “S” omitted in the figure) with a random environment $\alpha _E=\beta _E=\gamma _E=0$

**Fig. 3:**
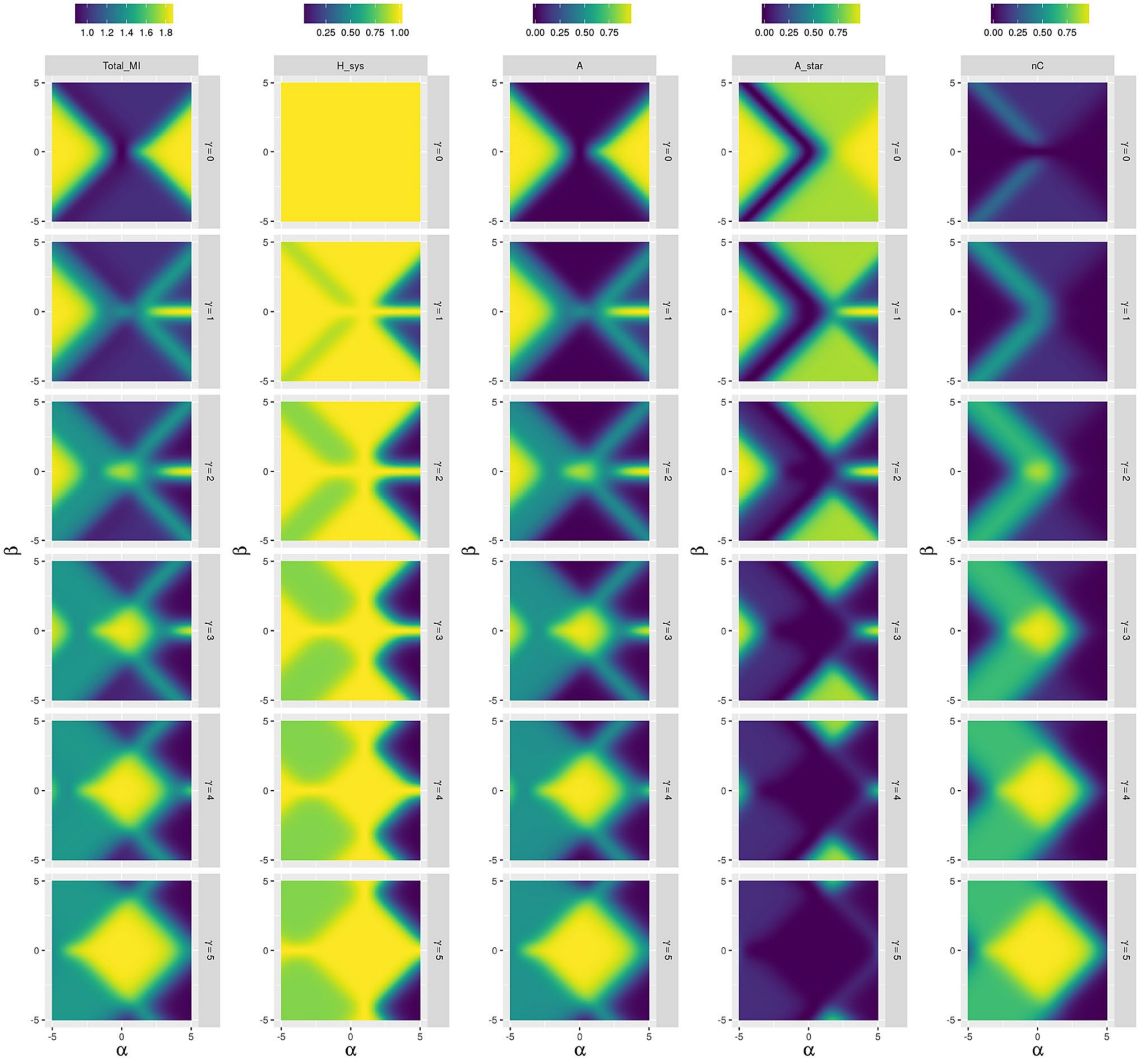
Mutual information between two time steps (Total_MI), Entropy of the system (H_sys), colonial (A) and organismal (A_star) individuality, and environmental determination (nC) for different values of $\alpha _S$,$\beta _S$, and for $\gamma _S$ with a correlated environment $\alpha _E=2 \quad \beta _E=\gamma _E=0$

We apply each individuality measure to this stochastic process. The results of this analysis are shown in Fig. 2 for a random environment and in Fig. 3 for an environment with memory. The panel sweeps through three coupling parameters for the systems state $s_{n+1}$: $\alpha _S$—the coupling parameter of the system state to its previous state $s_n$, $\beta _S$—the coupling parameter to the environment, and $\gamma _S$ the coupling parameter mediating the combined influence of the previous system and environmental states. When $\gamma _S=0$, we are not imposing any higher-order correlations on the time series.

When the value of $\gamma _S=0$, we detect colonial individuals as well as organismal individuals most readily at high values of $\alpha _S$ and $\beta _S$. When there are no higher-order interactions between system and environment then these two types of individual become indistinguishable in this parameter region and represent unique information in the system state. Both forms of individuality become more visible as more information is transmitted into the future. In the case of a non-random environment, the system can adapt to the environment and we observe high values of $A^\star$ together with low values of *A* and *nC* for high values of $|\beta _S|$ and low values of $\gamma _S$. Thus, the information flow from the environment into the system represented by high values of *nC* in the case of the random environment gets now internalized into the system.

As the value of $\gamma _S$ increases, the signatures of the organismal and colonial individuals diverge. Colonial individuals are most apparent at low values of $\alpha _S$ and $\beta _S$ where most of the information persistence derives from ongoing interactions between system and environment. Organismal individuals begin to disappear at high $\gamma$ as autonomy is lost. It is preserved only at high levels of $\alpha$.

The environmentally determined information transforms into colonial individuality at low $\gamma$ to becoming almost indistinguishable from it at high values of $\gamma _S$. This is because when the system and environment become strongly coupled, complementary information comes to dominate the signal, and the environment on its own becomes less predictive of the future state of the system.

The effect of $\gamma _S$ is to reduce the total entropy of the system (by creating systematic correlations and hence regularities in the information channel), and to reverse the pattern of total mutual information between successive time steps. This value is a minimum for low $\alpha _S$ and $\beta _S$ when $\gamma _S=0$ and a maximum when $\gamma _S=5.$

From the previous empirical example, we discern a process for identifying different forms of informational individuals in a more general setting. We find that system–environment distinctions increase in those parameters that increase independent memory ($\alpha , \beta$) when higher-order coupling is low. When this coupling increases, organismal individuals disappear and colonial individuals appear with reduced independent memories.

Let as assume that the transition parameters are held constant and we vary the system states. By systematically increasing the number of variables that we assign to the target system while reducing the environmental states, we can deduce whether this procedure leads to an increase in a suitable individuality measure.

If the expansion of the boundary of the system does not lead to an increase in information, then we have incorporated an environmental variable needlessly. In this way, individuals maximize their prediction of the future while minimizing their coding capacity. If individuality increases as we expand our system and environmental determination decreases, then we have grounds for the belief that we are capturing more of the individual by including more processes formerly treated as environmental.

Let us denote the original system with *S* and the part of the initial environment which becomes the system by $\Delta S$. The remaining environment should be denoted by $E'$.

- **E**nvironmental determination
  We get the two information flows
  $$\begin{aligned} nC&= I(S_{n+1};E_n|S_n) \\&= I(S_{n+1};E'_n \Delta S|S_n) \end{aligned}$$
  and
  $$\begin{aligned} nC'=I(S_{n+1} \Delta S_{n+1};E'_n| \Delta S_n,S_n) \end{aligned}$$
  Using some algebra we get
  $$\begin{aligned} nC'&= nC-I(S_{n+1};\Delta S_n|S_n)\\&\quad+I(\Delta S_{n+1};E'_n|\Delta S_n, S_{n+1},S_n) \end{aligned}$$
  The first term subtracts the information flow which is now internalized and the second term adds the flow which resided previously in the environment. Clearly the system becomes more closed if the former, now internalized flow, is larger than the latter.
- **O**rganismal individuality
  Let us start with the simpler measure $A^*$, the mutual information between subsequent states:
  $$\begin{aligned} A^*=I(S_{n+1};S_n) \end{aligned}$$
  and
  $$\begin{aligned} A'^*&= I(S_{n+1} \Delta S_{n+1};S_n \Delta S_n) \\&= A^*+I(\Delta S_{n+1};S_n|S_{n+1})+I(S_{n+1} \Delta S_{n+1};\Delta S_n|S_n) . \end{aligned}$$
- **C**olonial individuality
  $$\begin{aligned} A&= I(S_{n+1};S_n|E_n) \\&= I(S_{n+1};S_n|E'_n \Delta S_n) \\ A'&= I(S_{n+1} \Delta S_{n+1};S_n \Delta S_{n}|E'_n) \\&= A+I(S_{n+1} \Delta S_{n+1};\Delta S_n|E'_n)\\&\quad +I(\Delta S_{n+1};S_n|E'_n \Delta S_n S_{n+1}) \\&= A+I(S_{n+1};\Delta S_n|E'_n)+I(\Delta S_{n+1};S_n \Delta S_n|E'_n S_{n+1}) \end{aligned}$$

Both individuality measures can only grow or stay constant with increasing system size when information is available but they never decrease. Thus, they are not sufficient to detect the precise boundaries between individuals. In order to obtain precise boundaries we would need to impose a cost function—or regularizer—on system size to establish a threshold for termination. Our objectives here are not to find the optimal partition but present different informational “windows” on individuality.

## Implications of ITI

### Fundamental units and mechanism

Using an information-theoretic framework (ITI) applied to a stochastic process, we derived a number of principled quantities that capture forms and degrees of individuality. The approach has been somewhat formal as we have sought to provide a means for “detecting” or “perceiving” through an appropriate information-theoretic filter individuals in a variety of different evolutionary and ecological contexts. This is related to research that seeks to discover integrated spatiotemporal patterns for the purpose of discovering “agents” in a stochastic process (Biehl et al. 2016). It is also worth noting that the idea we can detect fundamental units in adaptive systems using an appropriate filter provides a second conceptual connection to physics beyond the thermodynamic connection outlined in A way forward section and discussed again in the next paragraph. Despite good theoretical reasons to expect the existence of particles beyond those predicted by the Standard Model, there is no direct empirical evidence BSM particles exist. To search for such particles, physicists are moving toward “model free” approaches, enhanced by machine learning (Collins et al. 2018), that allow detection of subtle correlations or anomalies in the data without making assumptions a priori about how the particles (presumably producing the anomalies) behave.

Questions of individuality connect to challenges related to explaining how functional space and time scales consolidate and new function emerges in biological systems (reviewed in Flack 2012, 2017a, b). This work suggests one driver of new function is the reduction in environmental uncertainty through the construction of dynamical processes with a range of characteristic time constants, described as nested slow variables. Slow variables are coarse-grained encodings of fast, microscopic dynamics. Slow variables provide better predictors of the local future configuration of a system than the states of the fluctuating microscopic components. As proposed in Flack (2017b) *maximizing uncertainty reduction through the computation of nested, coarse-grained slow variables, should be an organizing principle of adaptive systems* . This begs the question of how computations supporting regularity estimation get refined through learning and evolutionary dynamics and whether information processing is ever optimal, as some studies intriguingly suggest (Tkacik et al. 2012), and which provide support for the use of the information-theoretic formalisms supporting our individuality lenses.

### Levels of selection

The purpose of this paper has been to place the discussion of adaptive individuality on a solid logical and probabilistic foundation. In order to do so, we have taken a fair amount for granted, including the ability to make accurate measurements at arbitrary scales of granularity and over scales of time that are historically meaningful. We have also neglected to discuss those mechanisms that make heredity or transmission possible in the first place, in other words, robustness mechanisms that enable the error-free, or low error, transmission of information across generations. We have avoided discussing the specifics of the functional or selective benefits of hierarchical levels, concentrating on their identification. It is fair to assume that long-lived aggregates could develop the capacity to replicate and become a significant target for selection and hence a bona fide level at which selection operates, which for some is what is implied by biological individuality (Okasha 2006), in which case our approach could provide a means of identifying both pre-individuals (low autonomy) as well as fully fledged individuals (high autonomy). We discuss each of these topics in more detail below.

a *The partitioning requirement* In the previous discussion, we have “defined” the quantities, autonomy, closure and sufficiency, in terms of system and environment, but we have not provided any mechanisms that might generate a time series with appropriate values, or discussed how we might go about identifying the best system and environmental variables in the first place. Moreover, the choice of time scale will be instrumentally critical, as over very short or very long time scales we are unlikely to observe the regularities from which we seek to derive the individuality quantities: autonomy, sufficiency, and closure. It is our belief that few of these attributes (system and environment variables, time scales, etc) can be known in advance, and that it is precisely through the algorithmic determination of the individual that each will obtain relative support.
b *The robustness requirement* Further to identifying nested or hierarchical partitions, we also require some specification of the machine itself—the generator of the time series. This will be equated with parts of the individual and needs to possess some level of robustness or an error-correcting property. This is because individuality in adaptive systems often seems to be associated with adaptive mechanisms of homeostasis—mechanisms that monitor internal states and ensure that deviations are minimized. It is this self-preserving quality of the individual that allows us to make some useful discrimination between physical phenomena and biological ones, without exaggerating the dynamical differences.
c *The levels of selection* In many previous treatments of individuality, the idea that the individual has a special evolutionary status has been posited. This is presented in terms of levels of selection, where coarse-grained aggregates achieve a coordinated persistence property that now allows them to be treated as segregating, selective units. The most popular formalism for thinking about this process is presented in terms of the Price equation, which describes how the mean value of a trait changes as a function of the covariance in that trait and fitness, and the previous value of the trait. Of interest to us here is that the Price equation assumes some partition of trait values into groups and attempts to do this in such a way as to best capture the evolution of the mean value of the trait in the population. Assuming some true underlying structure and dynamics (see appendix of Nowak et al. 2010), the accuracy of the equation will depend on the choice of partition (Krakauer and Flack 2010b), and the ITI could provide such a principled platform for modeling.

## Future work

### Related formalisms

Before closing with a brief discussion of algorithms, a few comments about adjacent mathematical measures and approaches. Our approach is related to the concept of autopoiesis developed by Maturana (Maturana 1975) who emphasizes the “unity” of a network of processes engaged in self-production in terms of autonomy (Maturana 1980), and the idea of a Gestalt perception in which the figure is observed to be more than the sum of its parts and distinct from the parts of its grounding.

Another related body of work is the study of modularity network sciences. For static structures, there are reasonable definitions of modularity. Many of these definitions are associated with procedures for partitioning microscopic data into tightly bound groups, such as communities. For example, in networks quantitative modularity measures seek partitions of nodes and edges into sets that are statistically overrepresented in data when compared to an appropriate null model (Newman 2016). Developmental definitions of modularity, such as those applied to limb formation, or the appearance of body segments, also provide a window into individuality (Davidson et al. 2004) but they have not been presented in the form of quantitative measures for identification as they have in network science.

There is also a connection to the free-energy principle (FEP) as developed by Karl Friston and collegues Ramstead et al. (2018). Like the ITI, the FEP is built from first principles, moving forward from Schrodinger, and with the goal of explaining how adaptive systems resist decay and persist over time. It also stresses uncertainty reduction, but does through the lens of minimizing free energy. The FEP rests on the idea that adaptive systems will occupy a small bounded set of states within the total possible phase space. Furthermore, adaptive systems accomplish free energy minimization through construction of partitions that separate the organisms from its environment—in the FEP formulation this “filter” is a Markov blanket, which specify the conditional independence of internal and external states, with the internal states only perceiving the external states through the Markov filter.

### Algorithmic implementation

The last topic we discuss briefly is implementation of our measure on data. The ITI is mathematical formalism based on first principles for capturing information flow from the past to the future and which allows us to rigorously define a number of different forms of individuality. We have not provided an optimal algorithm for individuality-induction which we have left this for future work. Here, we do, however, note a few requirements.

A key empirical requirement is the careful measurement of a number of hypothesized individual attributes or properties over the course of time. For example, the abundance of organisms in a population; the genetic or phenotypic states of cells or tissues over time, the firing rates of neurons over time. In each case, we require a consistent time series of measurements in an appropriate coordinate frame (concentration, spatial position, firing rate, chemical concentration) that provide the input to our algorithms. It is our contention that many existing biological concepts (e.g., tightly coordinated replicators, developmental individuals), will be identified and become perceptible through this procedure. Many novel “individuals” might also be identified, including those at the societal level that are currently deprecated as derivative or epiphenomenal of lower level forms. And of great interest preadaptive organizations that emerge quickly relative to their own dynamical history and that experience a relatively long environmental history (i.e., self-organizing structures such as vortices that are picked up by the environmental determination measure).

## Appendix: Reflections on closure and sufficiency

We can think about an individual as a system that is a sufficient predictor of its own future. This implies that that $S_{n-1}$ does not add any information about $S_{n+1}$ besides the one already contained in $S_n$. Formally this reads as $I(S_{n+}; S_{n-1} | S_n) = 0$.

Note that by the Markovian structure of the system–environment interaction

$$\begin{aligned} I(S_{n+1}; S_n, E_n)&= I(S_{n+1}; S_{n-1}, S_n, E_n) \\&= I(S_{n+1}; S_n) + [ I(S_{n+1}; S_{n-1} | S_n) \\&\quad+ I(S_{n+1}; E_n | S_{n-1}, S_n) ] \\&= I(S_{n+1}; S_n) + I(S_{n+1}; E_n | S_n) \end{aligned}$$

Thus, $I(S_{n+1}; E_n | S_n) = I(S_{n+1}; S_{n-1} | S_n) + I(S_{n+1}; E_n | S_{n-1}, S_n)$ which means that informational closure implies sufficiency, i.e.,

$$\begin{aligned} nC=I(S_{n+1}; E_n | S_n) = 0 \implies I(S_{n+1}; S_{n-1} | S_n) = 0 \end{aligned}$$

Informational closure is therefore a stronger notion than sufficiency^2^ which allows the system to be influenced by the environment as long as this influence cannot be predicted from within the system.

### Considering longer histories

The above calculations can be generalized to longer histories:

$$\begin{aligned} I(S_{n+1}; S_n, E_n)&= I(S_{n+1}; S_{n-l}^n, E_{n-k}^n) \\&= I(S_{n+1}; E_{n-k}^n) + I(S_{n+1}; S_{n-l}^n | E_{n-k}^n) \\&= I(S_{n+1}; S_{n-l}^n) + I(S_{n+1}; E_{n-k}^n | S_{n-l}^n) \\&= I(S_{n+1}; S_{n-l}^n) + [ I(S_{n+1}; E_n | S_{n-l}^n) \\&+ \underbrace{I(S_{n+1}; E_{n-k}^{n-1} | S_{n-l}^n, E_n)}_{= 0} ] \end{aligned}$$

Thus, overall the same relationships between autonomy, closure and sufficiency are obtained

$$\begin{aligned}&I(S_{n+1}; E_{n-k}^n) + \underbrace{I(S_{n+1}; S_n | E_{n-k}^n)}_{A_k} \\&\quad = \underbrace{I(S_{n+1}; S_n)}_{A^*} + \underbrace{I(S_{n+1}; S_{n-l}^{n-1} | S_n)}_{\text{(non-)sufficiency }} + I(S_{n+1}; E_n | S_{n-l}^n) \\&\quad = A^* + nC \end{aligned}$$

### Sufficiency expansion and boundary detection

We can explore sufficiency a little more formally in terms of Markov conditions. We consider a system as self-sufficient if its dynamics are Markovian, i.e.,

$$\begin{aligned} I(S_{n+1};S_{n-m}|S_{n-1},\ldots ,S_{n-m+1})=0 . \end{aligned}$$

In the following we consider only the case $m=1$:

We get for the non-sufficiency $nS'=I(S'_{n+1};S'_{n-1}|S'_n)$ of the enlarged system

8 $$\begin{aligned} I(S'_{n+1};S'_{n-1}|S'_n)=I(S_{n+1} \Delta S_{n+1};S_{n-1} \Delta S_{n-1}|S_n \Delta S_n) \end{aligned}$$

Now we apply the chain rule:

$$\begin{aligned}&I(S_{n+1} \Delta S_{n+1};S_{n-1} \Delta S_{n-1}|S_n \Delta S_n)\\&\quad =I(S_{n+1} \Delta S_{n+1};S_{n-1}|S_n \Delta S_n) \\&\qquad +I(S_{n+1} \Delta S_{n+1};\Delta S_{n-1}|S_{n-1}, S_n \Delta S_n) \\&I(S_{n+1} \Delta S_{n+1};S_{n-1}|S_n \Delta S_n)\\&\quad =I(S_{n+1};S_{n-1}|S_n \Delta S_n)+I(\Delta S_{n+1};S_{n-1}|S_n \Delta S_n S_{n+1}) \\&I(S_{n+1} \Delta S_{n+1};\Delta S_{n-1}|S_{n-1}, S_n \Delta S_n)\\&\quad =I(S_{n+1};\Delta S_{n-1}|S_{n-1}, S_n \Delta S_n) \\&\qquad +I(\Delta S_{n+1};\Delta S_{n-1}|S_{n-1} S_n \Delta S_n S_{n+1}) \\&\text{ and } \\&I(S_{n+1};S_{n-1}|S_n \Delta S_n)\\&\quad =I(S_{n+1};S_{n-1}|S_n)+I(S_{n+1};\Delta S_n|S_{n-1} S_n) \\&\qquad -I(S_{n+1};\Delta S_{n} |S_n) \end{aligned}$$

which leads to

$$\begin{aligned} nS'&= nS-I(S_{n+1};\Delta S_{n} |S_n)+I(S_{n+1};\Delta S_n|S_{n-1} S_n)\\&\quad +I(S_{n+1};\Delta S_{n-1}|S_{n-1}, S_n \Delta S_n) \\&\quad +I(\Delta S_{n+1};\Delta S_{n-1}|S_{n-1} S_n \Delta S_n S_{n+1})\\&\quad +I(\Delta S_{n+1};S_{n-1}|S_n \Delta S_n S_{n+1}) \\ nS'&= nS-[I(S_{n+1};\Delta S_{n} |S_n)-I(S_{n+1};\Delta S_n|S_{n-1} S_n)] \\&\quad +I(S'_{n+1};\Delta S_{n-1}|S_{n+1} S'_n)+I(\Delta S_{n+1};S_{n-1}|S'_n S_{n+1}) \end{aligned}$$

We see that the only term which can lead to a decrease in the non-sufficiency is $I(S_{n+1};\Delta S_{n} |S_n)-I(S_{n+1};\Delta S_n|S_{n-1} S_n)$. It can be interpreted as the internalized information from $S_{n-1}$ to $S_{n+1}$.

The other two terms are always positive and related to new information flows through the environment—which are made possible by enlarging the system and which are not accounted for by *nS*.

## Footnotes

1 The terms of the partial information decomposition in Williams and Beer (2010) were derived from a set of very general axioms. These axioms, however, do not define concrete measures for the specific term. One needs an additional definition for one of the four terms. The original proposal by Williams and Beer (2010) was criticized as counterintuitive and several other proposals have been made, such as (Harder et al. 2013; Bertschinger et al. 2014; Finn and Lizier 2018; James et al. 2017), albeit no consensus has yet been reached. In this paper, we will use the decomposition as a conceptual framework to interpret classical information-theoretic quantities.
